# Simple tricks for improving pattern-based information extraction from the biomedical literature

**DOI:** 10.1186/2041-1480-1-9

**Published:** 2010-09-24

**Authors:** Quang Long Nguyen, Domonkos Tikk, Ulf Leser

**Affiliations:** 1 Knowledge Management in Bioinformatics, Department for Computer Science, Humboldt-Universität zu Berlin, Germany; 2 Department of Telecommunications and Media Informatics, Budapest University of Technology and Economics, Hungary

## Abstract

**Background:**

Pattern-based approaches to relation extraction have shown very good results in many areas of biomedical text mining. However, defining the right set of patterns is difficult; approaches are either manual, incurring high cost, or automatic, often resulting in large sets of noisy patterns.

**Results:**

We propose several techniques for filtering sets of automatically generated patterns and analyze their effectiveness for different extraction tasks, as defined in the recent BioNLP 2009 shared task. We focus on simple methods that only take into account the complexity of the pattern and the complexity of the texts the patterns are applied to. We show that our techniques, despite their simplicity, yield large improvements in all tasks we analyzed. For instance, they raise the F-score for the task of extraction gene expression events from 24.8% to 51.9%.

**Conclusions:**

Already very simple filtering techniques may improve the F-score of an information extraction method based on automatically generated patterns significantly. Furthermore, the application of such methods yields a considerable speed-up, as fewer matches need to be analysed. Due to their simplicity, the proposed filtering techniques also should be applicable to other methods using linguistic patterns for information extraction.

## Introduction

As the amount of biomedical knowledge recorded in texts is steadily increasing, it is more and more important to develop efficient techniques to automatically discover new, hidden, or unsuspected knowledge in large text collections [1]. Many text mining (TM) methods have been developed in order to achieve this goal [2]. TM aims to discover and extract knowledge from unstructured text, making use of natural language processing (NLP) tools, such as tokenizers, morphological analyzers, part-of-speech-taggers (POS-taggers), and syntactic or deep parsers to process texts [3]. TM in the biomedical field mostly focuses on two tasks: Named Entity Recognition (NER), i.e., the task of recognizing various types of biological entities (genes and proteins, chemicals, diseases etc.), and Relation Extraction (RE), i.e., the task of identifying relations among those entities.

The classical baseline approach to relation extraction is to only use information about the co-occurrence of entities in the given scope (sentence or abstract; [4,5]). Using co-occurrence is popular, because it is easy to implement and allows for efficient processing of huge amounts of texts. However, co-occurrence-based approaches fail to characterize the biological meaning of an interaction (e.g., inhibition or stimulation, directionality) and, more importantly, also generate many false positives because they cannot distinguish positive from negative pairs. The second important class of approaches is based on machine learning. Here, a statistical model is learnt from a set of positive and negative examples and then applied to unseen texts. In general, machine learning-based methods to RE perform very well for any task where sufficient and high-quality training data is available [6,7]. However, their major drawback is this need for training data, which, in the Life Sciences, is only available for the most common tasks and very costly to produce. Furthermore, machine learning-based methods are prone to overfit to the training corpus, which makes results of evaluations very hard to extrapolate to other texts [8].

A third class of relation extraction methods is based on pattern matching. Pattern-based approaches build on annotated text fragments (the patterns), where words/phrases are labelled with linguistic information, e.g. POS-tag, word lemma, or syntactic information. Those patterns are matched against linguistically annotated text to detect relationships. Patterns can either be learned from examples [9,10] or can be defined manually [11-13]. Pattern-based systems have shown good results in many areas of biomedical text mining, such as extraction of PPI [10,13] or the reconstruction of pathways [14].

Systems based on manually-defined patterns typically use relatively few patterns, leading to high precision but low recall [15,16]. In contrast, systems that learn patterns automatically often produce thousands of patterns and exhibit a better recall, but often at the cost of a decrease in precision. To circumvent this penalty, one may try to improve a given set of patterns using some optimization criteria. As an example, SPIES [10] proposed a pattern set optimization method based on the minimum description length (MDL) principle from information theory [17]. Starting from the set of all patterns retrieved from the training set, iteratively two patterns are selected for merging such that (1) a merging criterion is maximized; (2) the merged pattern does not violate some task-specific heuristic constraints. This procedure has increased the F-score for PPI extraction by 6.7%, but the MDL-based algorithm is quite complex and rather cumbersome to adapt to a new problem.

In this paper, we propose much simpler ways of improving pattern-based relation extraction. We focus on simple filtering techniques that take into account the complexity of the pattern and the complexity of the texts the patterns are applied to. Our ideas are based on the observations that (1) very oftenmost of the patterns in an automatically learned pattern set are used rarely, that (2) complex patterns tend to produce more false matches, and that (3) complex or ambiguous phrases in the text tend to produce higher error rate.

We evaluate our ideas using the corpus of the BioNLP'09 Shared Task (hereafter BioNLP task) [18] and the pattern-based text mining system Ali Baba [19]. While earlier shared tasks (such as BioCreative II, [20]) have dealt with general PPI, the BioNLP task focused on a set of more specific molecular events, such as gene expression, protein catabolism, or positive regulation and negative regulation. We hypothesized that a well-performing relationship extraction system like Ali Baba would perform well also on these tasks, because (a) all targeted events encompass the relationship between at least two constituents (either entities or trigger words), and (b) 95% of all annotated events are fully contained within a single sentence [21].

However, its initial performance was rather poor. However, by using simple text and pattern filters, we were able to improve F-scores considerably. For instance, the unfiltered pattern set led to an F-score of only 24.8% on gene expression events, but the score rose to 51.9% after filtering. Although we evaluated our ideas only using Ali Baba, we want to stress that our filters are defined in such a way that they also should be applicable to other tasks and to other pattern-based extraction methods.

## Methods

### Ali Baba

Ali Baba is a tool which automatically parses PubMed http://www.ncbi.nlm.nih.gov/pubmed/ abstracts matching a given user query. The set of matching abstracts is further processed using a dictionary-based NER (for genes, diseases, cell lines, species, drugs, tissues etc.) and a pattern-based algorithm for the detection of protein-protein interactions (see below). Dictionaries are compiled from several biological databases (UniProt http://www.uniprot.org/, NCBI Taxonomy http://www.ncbi.nlm.nih.gov/Taxonomy/, MesH http://www.ncbi.nlm.nih.gov/mesh/, KEGG http://www.genome.jp/kegg/, Drug-Bank http://www.drugbank.ca/ etc.). For matching, all entity names are converted into regular expressions that also match in the presence of slight linguistic variations (plural etc.). The resulting network of entities connected via relationships is visualized as a graph, thus presenting a quick overview over the most important information contained in the abstracts [19].

### Pattern-Based Information Extraction with Ali Baba

Ali Baba uses a pattern-based approach to extract relationships among entities. To this end, it learns typical linguistic representations of relationships and encodes them in language patterns. Patterns are task-specific, i.e., they depend on the type of relationship that should be extracted, and learning is performed in three steps. Input to the learner is a set of positive examples, i.e., a set of tuples where each tuple consists of entities that are in the sought-for relationship. For instance, for detecting PPI, the input must be a list of interacting proteins.

In the first step, Ali Baba collects a list of sentences that contain both elements of any of the input tuples plus a trigger word taken from a list of words specific to the type of relationship being searched. This step may be performed, for instance, by searching a text database such as PubMed. In the second step, all those sentences are linguistically annotated by adding word stems and part-of-speech tags. Next, the annotated sentences are reduced to their 'core', i.e., the minimal token span containing all elements of the tuple, the trigger word, and a neighbourhood of configurable size. All matched entities are replaced with a placeholder. From those core phrases, Ali Baba generates a set of patterns, each of which consists of three layers: the core phrase itself, a stemmed version of the phrase, and its sequence of POS tags (see Table 1 for an example). In the third step, the system clusters all initial patterns based on a similarity measure that builds upon a pair-wise multi-level alignment of core patterns (see below). For each cluster, a multiple sentence alignment is computed and condensed into a profile per cluster, capturing the relative frequencies of tokens, stems and POS tags for each position of the pattern. This last step is optional; when initial patterns are directly used for matching, we usually observe very good precision but low recall. After clustering, precision usually drops, but recall increases considerably as the merged patterns are more general than the initial ones. Details on the entire process may be found in [22].

**Table 1:** A simplified example of an initial pattern of the Ali Baba system (derived from a sentence from document 1520341 of the BioNLP task).

Token layer	CD19	protein	is	expressed
**Word stem layer**	PTN	protein	be	express

**POS layer**	PTN	NN	VBZ	GEE

PTN indicates protein name; VBZ, verb in present tense; NN, singular noun; GEE, gene expression event trigger.

Ali Baba matches patterns against sentences using (as for the clustering) multi-layer sentence alignment, a procedure conceptually similar to sequence alignment, but performed on tokens, stems, and POS tags, not on single characters. The matching allows for differences between the pattern and a sentence in the same manner as sequence alignment introduces insertions, substitutions, deletions, and mismatches between DNA or protein sequences. Furthermore, the decision whether or not a term of a pattern matches a term of a phrase is not binary, but the specific term pair is assigned a score by looking up a substitution matrix [9]. For each layer there is a dedicated substitution matrix and the overall score for each pair of terms is a linear combination of all scores for the different layers. Increasing or lowering the threshold for this score tunes the method towards higher precision or higher recall, respectively.

### BioNLP'09 Shared Task

To focus efforts on the general problem of event extraction, we decided to use the corpus provided by the international BioNLP'09 Shared Task http://www-tsujii.is.s.u-tokyo.ac.jp/GENIA/SharedTask/. This task concerns the detection of complex events in biomedical text, such as gene expression, transcription, or protein catabolism. It consisted of several subtasks. The objective of the Task 1 was to identify events with their types, trigger expression in the text, and primary theme and cause arguments. The competition provided crisp task definitions, data sets for training, development, and testing, an evaluation script for the training and the development sets, and an online tool for evaluation on the test set.

In all data sets, the proteins that took part in the events were annotated in the text; i.e., NER was not part of the task. Note that the performance of event recognition would certainly drop significantly when NER were part of the evaluation procedure (see Kabiljo et al. [23] for a quantification of this effect on PPI extraction). For the training and development sets, the participants were also provided with the gold standard for events and their respective theme(s).

The data sets were prepared based on the GENIA event corpus [24]. The data for training and development sets were derived from the publicly available part of GENIA, while the test data were taken from an unpublished part. Table 2 shows statistics of the different data sets.

**Table 2:** Statistics on the three data sets provided by the BioNLP task.

	Training	Development	Test
Abstracts	800	150	260
Sentences	7,449	1,450	2,447
Words	176,146	33,937	57,367
Gene expression events	1,738	356	722
Protein catabolism events	111	21	14
Transcription events	576	82	137
Phosphorylation events	169	47	135

### Application of Ali Baba to the BioNLP Shard Task

We applied Ali Baba's algorithm for pattern generation and matching to four event types of the BioNLP task (gene expression, phosphorylation, catabolism, transcription). We will explain the process using as example the gene expression task, which also has the highest number of examples in the corpus (1738 samples in training, 356 in development, and 722 in test). Application to the other event types followed the same strategy. Recall that proteins are already annotated in the BioNLP'09 corpus and NER thus was not part of the evaluation. If not indicated otherwise, we report on event level approximate span matching results (see the description in [18]) in terms of precision, recall and F-score.

Ali Baba's pattern learning method was applied with slight modifications. We decided to use only the information provided by the BioNLP organizers. The main reason for this was the expected high level of noise when we would have searched PubMed for positive examples, which, for the gene expression event type, would consist only of a gene name and a trigger word (in contrast to tasks such as PPI extraction, where positive examples would be identified by two protein names in addition to trigger). Therefore, we first collected all sentences that describe a gene expression event from the 800 training documents. Linguistic annotation and reduction to core phrases were performed as described above. We omitted the third step in Ali Baba's pattern learning (clustering of patterns) because our aim here was to study a different way of improving extraction performance, i.e., by filtering patterns and not by merging them. Furthermore, pattern clustering is highly specific to Ali Baba; including this step would make our results less applicable to other pattern-based relation-ship extraction method.

Result from applying Ali Baba in this way on development and test corpus are shown in Table 3 (first line). The results show that there is much room for improvement; especially precision values are very low.

**Table 3:** Effect of filtering on combined training data (cross-validation folds from development and training corpus) and on the held-back test data set.

	Development (per split)					Test		
		
	# patterns	Aver. pattern length	Precision	Recall	F1	Precision	Recall	F1
Baseline	590	8.93	24.7	49.2	32.9	17.2	43.9	24.8

Split 1	50	5.34	65.6	51.8	57.9	64.7	42.7	51.4
Split 2	50	4.86	78.1	52.3	62.6	63.0	37.8	47.3
Split 3	60	4.68	67.6	52.9	59.3	60.9	42.5	50.1
Split 4	40	5.02	67.7	49.5	57.2	66.6	36.7	47.3
Split 5	50	4.80	63.7	48.7	55.2	64.2	40.7	49.8

Union of patterns	104	5.65				58.2	46.8	51.9
Best 90	90	5.66				59.7	45.1	51.4
Best 80	80	5.75				64.8	37.7	47.6
Best 70	70	6.01				69.4	26.7	38.6
Best 60	60	6.17				60.0	10.0	17.1

Results of the winner of the shared task [21]						78.5	69.8	73.9

See the definition of splits in text in Results (Evaluation of Test Data)

### Filtering Patterns

Based on the baseline results, we performed a detailed error analysis. This analysis revealed that the bulk of false positives stem from a surprisingly small number of error types. Furthermore, these types of error mostly can be removed by filtering certain patterns or by disregarding certain sentences. In the following sections, we present several such filters and their effect on the performance in detail. We also report on some alternative directions we followed but which did not improve performance significantly. Finally, we analyze the overall performance when several filters are combined.

We consider improvements based on two main strategies. The first is a trigger-word-based pattern filter. Trigger words are specific tokens in a sentence which indicate the occurrence of an event. Inspecting the trigger words for gene expression in the training data reveals that many textual expressions are triggers in some case but not in others (see Table 4). For instance, only 44% of the occurrences of the token 'expresses' are trigger for a gene expression event, while the remaining 56% are not triggers for any annotated event. Another example is that one-third of the occurrences of the token 'overexpression' appear as gene expression event trigger, while the other two-thirds are triggers of other types of events. The second strategy to improve the quality of the pattern set is filtering patterns based on some of their properties. Filtering is based on the idea that not all patterns are equally good. We performed a separate evaluation of every individual pattern which allowed us to identify those patterns that decrease performance, and analyzed those patterns to find commonalities among them.

**Table 4:** Evaluation of trigger words for the gene expression event.

Trigger word	FP	TP	Occurrence	Hit rate
co-expression	0	2	3	0.7
coexpressed	0	4	6	0.7
nonexpressing	0	2	3	0.7
expressing	1	44	75	0.6
expressed	25	136	232	0.6
express	15	43	81	0.6
production	6	150	298	0.5
co-transfections	1	1	2	0.5
resynthesized	0	1	2	0.5
expression	106	873	1768	0.5
produce	2	16	33	0.5
expresses	0	4	9	0.4
produces	0	2	5	0.4
overexpression	53	27	80	0.4

FP: number of cases where the trigger word is trigger of other event types. TP: number of cases where the trigger word is a gene expression trigger. Occurrences: Total number of occurrences in corpus. Hit rate: see definition in text in Results (Trigger Word Filter).

## Results

### Pattern Score Threshold

We first ran an experiment to test the applicability of the general algorithm behind Ali Baba on the BioNLP task data. As stated in Methods, pattern matching in our system generates a score for each pattern against each sentence. A threshold on this score determines which matches are considered as positives. Additional file 1 depicts the effect of changing this threshold. As expected, raising the threshold leads to higher precision at lower recall, while lowering it has the opposite effect. In our experiments, we decided to set the threshold such that the F-score was optimal on the development set for the baseline implementation.

### Trigger Word Filter

**Figure 1:**
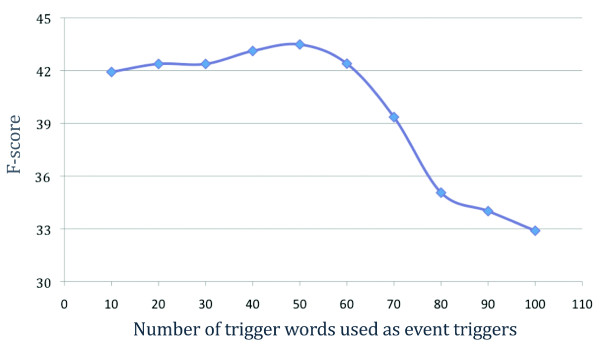
**F-scores on gene expression event extraction when considering only the *k *best trigger words (*k *= 10,...,100) sorted by decreasing hit rate**.

We use the hit rate (HR) of a trigger word for its assessment. For a given trigger word, the HR is the number of occurrences of the word in a sentence with the respective event type divided by the total number of occurrences in the gold standard annotations (see Table 4). We investigated all trigger words from the training and development sets and found that triggers with low HR degrade precision and were also found to also produce more event type mismatches (e.g. positive regulation instead of gene expression) than other patterns. We therefore sorted all the 133 trigger words according to their HR in a descending order and cumulatively evaluated the impact of using only the subsets of 10 to 100 event triggers having the best HRs. Results are shown in Figure 1.

Based on these results, we fixed a set of 50 event trigger words that yielded the best F-score on the development corpus. Note that all triggers words in this set are single-word triggers and either verbs, nouns, or adjectives. The impact of this simple filter is already quite large (see Table 5). On the development data, precision increased by 60% (from 24.7% to 39.7%) compared to the baseline, while recall dropped only minimally (from 49.2% to 48.0%). F-score grew from 32.9% to 43.5%.

**Table 5:** Evaluation of gene expression event extraction using different combinations of filters.

Filter	Precision	Recall	F1
No filter	24.7	49.2	32.9
Trigger Word	39.7	48.0	43.5
Pattern Length (exactly 4 token)	51.0	29.8	37.6
Pattern lengths (≤ 4 token)	56.2	43.5	49.0
Pattern Performance (top 50 pattern)	50.0	48.3	49.0
Trigger Word + Pattern Length	65.6	39.3	49.2
Trigger Word + Pattern Performance	77.4	46.3	58.0

### Pattern Length Filter

Additional file 2 depicts the length distribution of all patterns extracted from the training data. The length of most patterns is between 2 and 19 tokens, with most being between 4 and 8 tokens long. Inherently, Ali Baba's pattern matching algorithm favours longer patterns since those aggregate a score over more token matches. Consequently, even quite dissimilar phrases may still produce overly positive scores, if some tokens with large weights coincide. This is an intrinsic property of the alignment algorithm that is not easily circum-vented by normalization by pattern length. However, we hypothesized that simply using only shorter patterns could already reduce the error rate.

**Figure 2:**
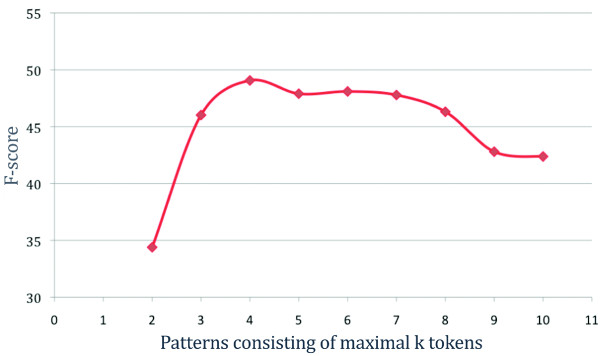
**F-scores on gene expression event extraction when using only pattern consisting of maximal *k *tokens**.

We evaluated this hypothesis via two experiments: First, we partitioned all patterns into subsets of the same length and evaluated every subset separately on the development corpus. We found that patterns consisting of four or five tokens yielded the best F-score, both 37.6, which is 14% better than the baseline, while long patterns perform considerably worse (see Additional file 3 and Additional file 4). As expected, the precision of each subset was boosted remarkably, while the recall of each evaluation run was much lower than when using all patterns. To prevent this decrease of recall, we then created subsets of patterns according to their maximum number of tokens. The evaluation results (see Figure 2) show that the set of patterns with the maximum length of four (141 patterns) yields the best F-score.

The impact of the pattern length filter on the performance is shown in Table 5. The filtering yields a large improvement in precision (more than doubling) together with a modest decrease in recall (by 12%). The F-score increased from 32.9 to 49.0 when compared to the baseline.

### Pattern Performance Filter

The previous experiment evaluated classes of patterns based on their length. We then studied the individual performance of each of the 590 initial patterns. As usual, patterns were extracted from the training data while evaluation was performed on the development data to minimize overfitting. Because recall of an individual pattern is naturally low, we concentrated on their precision. To this end, we ran Ali Baba's matching phase once for every pattern of the entire pattern set and recorded the precision of each pattern.

**Figure 3:**
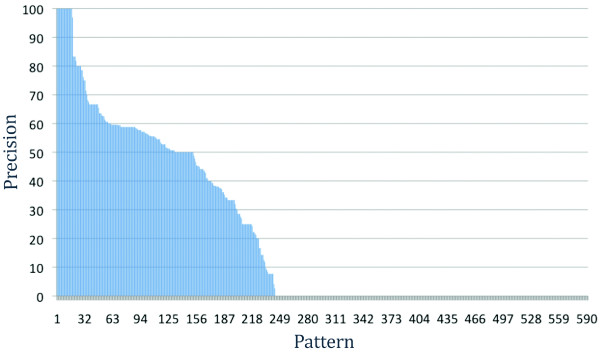
**Precision of 590 patterns on extracting gene expression event extraction**. Patterns are sorted by decreasing individual precision.

**Figure 4:**
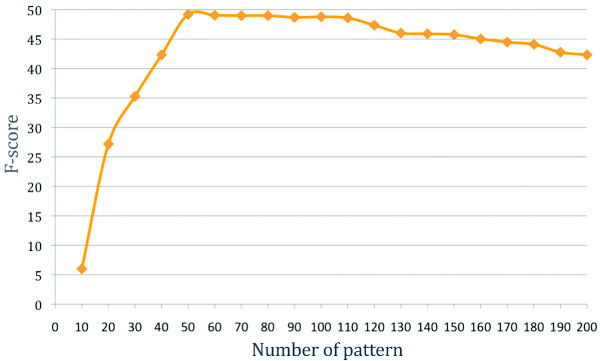
**F-score of gene expression event extraction when considering subsets of 10 to 200 best patterns sorted by decreasing precision**.

Figure 3 shows that the differences are tremendous, covering the entire range from 100% to ~8% precision. Additionally, we found almost 400 patterns that did not match any sentence in the development corpus. Ignoring those patterns may lower the recall on a larger corpus but would increase the precision and also speeds up the processing time. We then used the same method as in the pattern length filter to evaluate subsets of patterns defined on their individual precision. That is, we reran the experiment on subsets of 10 to 200 best patterns (see Figure 4).

The best F-score was achieved using the 50 best patterns. Table 5 compares the performance before and after filtering. The use of the pattern performance filter doubles the precision while it decreases the recall by a mere 2%, when compared to the baseline. In addition, the filter drastically decreases the runtime, from 43 minutes to 3.5 minutes, as fewer patterns need to be matched against sentences.

### Protein Number Filter

We also experimented with another filter assuming that sentences that contain many proteins produce more errors, because it should be more difficult for the matching algorithm to find the right assignment from trigger words to entities. To test this hypothesis, we sorted all sentences from development corpus according to the number of proteins they contained, and partitioned the corpus into 8 smaller corpora, where each corpus contained all sentences with exactly *k *proteins (*k *= 1,...,8). We evaluated each partition separately. Contrary to our expectation, the performance on the different partitions did not differ significantly (data are not shown).

### Combining Filters

After inspecting the performance of each filter separately, we investigated whether their combination could further improve the performance. Table 5 summarizes our results for using combined and individual filters on the development data. The best performance was achieved by the combination of the pattern performance filter and the trigger word filter. These two filters together raised precision from 24.7% to 77.4%, i.e., the precision has tripled. At the same time, recall decreased only slightly, from 49.2% to 46.3%. The F-score almost doubled, from 32.9% to 58.0%.

### Evaluation on Test Data

For the evaluation on the test data set we used the union of training and development data as training corpus. Measuring the performance on a data set different from the training data is essential to prevent overfitting, because training and evaluating a statistical model on the same (or overlapping) data may lead to the overestimation of the method's performance [25]. Note that the ground truth of event annotations on the test data is not available, but one can measure the performance of a method using the submission system of the BioNLP task. This opportunity is limited for one submission per user per day to prevent the server overload and fine-grained optimization against the test set. Since our pattern filtering methods presented so far require a development set against which the patterns can be optimized - which is not possible on the test set - we proceeded as follows to select a set of good patterns for the test data.

We created several train-development splits from the combined training corpus of 950 abstracts, optimized the pattern sets of each split using our pattern filters and then combined the optimized pattern sets. For the train-development splits, we randomly re-sampled the combined training corpus five times. In each split, we randomly selected 800 abstracts for training and used the remaining 150 abstracts for development. For each training set, we individually computed the optimal number of patterns with respect to F-score measured on the corresponding development set (as described in the Pattern Performance Filter Section). Then, we applied the pattern performance filter and the pattern length filter. In the first block of Table 3, we report on the performance of the five optimized pattern sets measured on the corresponding development set and on the test set.

All the five optimized pattern sets consist of 40 to 60 patterns and have an individual F-score between 55.2 and 62.6 (mean 58.46, standard deviation 2.48). Their performance on the test data lies between 47.3 and 51.4 (mean 49.18, standard deviation 1.63), and is thus approximately 10% lower on average than the performance on the development data. The difference can mostly be attributed to a drop in recall, showing that the different corpora have different characteristics; it comes as no surprise, as patterns, when learned and tuned on such small corpora as available for the BioNLP task, inherently overfit. What is more interesting is that the precision remains fairly stable, which is very encouraging as it shows that well-performing patterns perform well independent of the corpus from which they are obtained.

Additionally, we combined all distinct patterns from all five experiments into a set of 104 unique patterns. We sorted the patterns according to their precision achieved on the corresponding development split. Then, we took the best 60, 70, 80, 90, and the full set of 104 patterns and evaluated each set on the test corpus. Results are also shown in Table 3.

Again, one can see that recall of patterns is rather corpus-specific and that using only the best 60 patterns from the different experiments results in a very low recall on the test data. This implies that in each split, a large fraction of the best-performing patterns (in terms of precision) is highly corpus-specific and produce much rarer matches on the test set. This effect disappears gradually when more patterns are considered. Also as before, precision remains fairly stable (with an outlier for 70 patterns) and roughly lies in the same range as results for any of the individual experiments. The best F-measure is achieved when using all 104 unique patterns.

Recall that the gold standard annotation on the test data currently is not directly available. Evaluation on this data set is only possible by sending-in an annotated corpus to the online submission system of the shared task, and these evaluations are limited to one per day and person. Therefore, it was not feasible to find the optimal set of patterns on this data set, as this would require separate runs for each pattern (this would have taken 590 days). In turn, this means that the results practically simulate the case of applying our method on unseen text: selecting the optimal pattern set on training data and applying it on the new (test) data. Accordingly, it is not clear that the set of 104 patterns is really the best, but it is reassuring that it produces the best results from all runs we performed. Further optimizing the number of selected patterns would only make the results more corpus-specific and less applicable to unseen text.

### Results on Other Event Types

We also applied the trigger word and pattern performance filter to three other event types of the BioNLP shared task: phosphorylation, protein catabolism, and transcription. Evaluation method was the same as for gene expression. To be able to reuse the same methods, we had to modify slightly the setting for the phosphorylation task, because this event type may encompass three slots instead of only two (as the other three event types): protein, trigger word, and phosphorylation site. However, detecting the site is very simple. We tagged all mentioning of sites using regular expressions and used the pattern matching algorithm only for detecting and associating protein and trigger word. Since this deviates from the standard procedure, we do not compare our results with those of the task, but only focus on the improvements achieved by our filtering. Finally, trigger word filtering was not tested on phosphorylation as the number of trigger words is very small for this event type. On the test data, we only evaluated the best filter according to the development data. Results are shown in Table 6; the table also contains the results achieved by the best solution in the BioNLP [21].

**Table 6:** Results for the extraction of event types other than gene expression (phosphorylation, transcription, and protein catabolism) on development and test data sets.

	Precision	Recall	F1
**Protein catabolism - dev data**			
Baseline	3.6	42.8	6.6
Trigger Word Filter	3.2	38. 1	5.9
Pattern Performance Filter	61.5	38.1	47.0
**Protein catabolism - test data**			
Baseline	1.8	50.0	3.4
Pattern Performance Filter	50.0	14.2	22.2
Best in shared task	66.6	42.8	52.1
**Transcription - dev data**			
Baseline	3.8	26.8	6.7
Trigger Word Filter	4.4	21.9	7.4
Pattern Performance Filter	18.1	24.3	20.8
Trigger Word + Pattern Perf	35.3	20.2	25.5
**Transcription - test data**			
Baseline	4.1	29.2	7.2
Trigger Word + Pattern Perf	23.8	11.2	15.3
Best in shared task	69.2	39.4	50.2
**Phosphorylation - dev data**			
Baseline	2.4	35.6	4.6
Pattern Performance Filter	40.0	34.0	36.7
**Phosphorylation - test data**			
Baseline	3.0	49.2	5.7
Pattern Performance Filter	72.7	47.4	57.4
Best in shared task	91.2	76.3	83.0

The table also reports on winner's performance in the shared task on the test data [21]. Best results for phosphorylation are omitted as these are not directly comparable (see text for expla ation).

The data shows that filtering drastically improves the performance in all cases. The improvement in F-measure is up to tenfold. Note that the numbers of examples for these event types are much smaller than for gene expression, which explains the general low performance of the baseline on the test data. However, pattern filtering considerably improved performance also on test data.

## Discussion

We described simple techniques for filtering sentences and patterns to improve pattern-based relationship extraction with an automatically learnt pattern set from the biomedical literature. A proper combination of the trigger word filter and the pattern performance filter has a major effect on the performance both on the training and on the test data for all event types we considered. For gene expression events, we were able to improve the precision from 24.7% to 77.4% and the F-score from 32.9 to 58.0 on the development corpus. The decline in recall was comparably small (from 49.2% to 46.3%). The filters also achieved a significant improvement when evaluated on the test data set, where the precision grew from 17.2% to 58.2% and the F-score from 24.8 to 51.9. Evaluation on three other types of events (phosphorylation, catabolism, and transcription) often showed even larger improvements in precision and F-measure.

In general, we expected that our approach would yield higher precision but lower recall. The evaluation confirmed this expectation for almost all event types and both data sets. Filtering inherently decreases recall as it only suppresses potential match situations, but does not add new ones: thus, only FPs can be avoided, but no new TPs can be added. On the other hand, if applied properly, filtering should increase precision by avoiding erroneous patterns or difficult sentences which have a higher chance of producing FPs. The trick is to find the right balance between decrease in recall and increase in precision. In this work, we learned optimal thresholds in terms of the hit rate of trigger words and length or performance of individual patterns by using a development corpus. However, we found that the learned parameters seem to be reasonable, as they led to considerable improvement also on the test data sets.

It is surprising that, only for gene expression, even recall improved on the test data. How-ever, this behaviour can be explained by the fact that, in the baseline, some patterns produced false positives but high scores in the matching algorithm of Ali Baba, thus overruling other patterns which would have found the right hit but produced lower scores. These patterns were removed from the filtered pattern set. This effect was not observed for the other event types, very likely because for those the number of patterns is much smaller. Our results show that it is possible to achieve good result using only a small set of 50 patterns (containing in average 4.82 tokens), which also makes the extraction very fast. We observed a similar effect in [26], where, for each sentence, we first sorted all patterns according to a rough estimate on their expected maximal score (based on q-gram frequencies in the pattern and the sentences) and only applied the best *k *patterns for the actual extraction. This yielded a 100-fold increase in extraction speed at ~3% worse F-measure. The results of our current work are orthogonal: here, we focus on improving extraction quality. Patterns are filtered globally using optimization on a development corpus instead of being selected heuristically on a per-sentence basis as in [26]. In our current work, the observed speed-up is more of a side-product.

We are aware of only a few other works that evaluated filtering of patterns in pattern-based IE. In the Life Sciences, the SPIES project [10] used a pattern filter based on the Minimum Description Length Principle (MDL) for PPI extraction (see Introduction). The effect they report is much smaller than the one we observed, probably because their performance without filtering was already much higher than in our case, leaving less room for improvement. This higher performance can be explained by the fact that the SPIES system was evaluated only on a corpus that contained no sentence without a positive example. Filtering by trigger-word-based pattern evaluation was also performed by Cohen et al. [16] and by Buyko et al. [27], specifically for the BioNLP task. Cohen et al. [16] order trigger words by frequency and keep only the top 10-30% (depending on the event type) for pattern generation. In contrast to our filter, their solution did not take into account the ambiguity of trigger words across event type, but they allowed for some linguistic variations. Buyko et al. [27] tested three variations of treating trigger-word ambiguity, i.e., by frequency, by TF*IDF score, and by importance (taking into account relative frequencies in the different event types). The authors, in accordance with our results, report that the filter considering the frequencies in different event classes performed best. Note that they used these scores as input to a machine learning approach to relationship extraction, while we work with pattern matching. A system outside the Life Science' domain is DARE [28]. DARE uses so-called seed relations, specified manually, as initial patterns. These are applied on a large corpus to generate new examples that, iteratively, are used as new seeds again. All patterns are scored according to internal and external properties before being used as new seeds. In contrast to our proposals, the pattern scoring method is highly specific to DARE and not easily transferred to other approaches.

The overall performance of our system cannot compete with the best approaches reported for the BioNLP task (see Table 3, Table 6, and [18,21]). However, we remark that we applied Ali Baba's method practically without any tuning to the task, while most high-scoring systems were developed specifically for this competition. It would be interesting to see how well they perform on other corpora and other tasks. Often, tuning for a specific corpus yields systems that perform considerably worse on other data; for instance, the OpenDMAP system, using a set of manually created patterns, performed best in the BioCreative II PPI task [29], but was found to perform worst (compared to 5 other systems, including co-occurrence as baseline) on each of five other corpora [23]. This system also achieved exceptionally good precision results on the BioNLP task (with a new set of manually created rules) [16], with F-scores being sometimes better and sometimes worse than our results using filtering.

Another important difference between Ali Baba and the top-performing systems in the BioNLP task is the speed of extraction. All top-scoring systems in the task used dependency parse trees as their primary input. Such an approach requires high-cost computer equipment when applied to sizeable text collections and not only a couple of hundred sentences. For instance, Björne et al. [30] estimate that it would take ~230 days to parse all sentences in PubMed which contain at least one protein name, using the Charniak-Lease parser [31]. In contrast, Ali Baba only requires sentences to be POS-tagged, which can be accomplished in less than a day on a modern quad-core machine. Whether or not systems using only POS tags can - in general - perform equally well as those using dependency parses is a question that is not yet decided; in our recent evaluation on the performance of PPI extraction systems on five PPI corpora using three different evaluation strategies [8], the shallow linguistic kernel based system [32], which uses only POS tags and tokens, performed almost equally well as the best systems using dependency parse trees.

## Conclusions

We studied the effect of several simple pattern selection techniques on the performance of pattern-based relation extraction methods in biomedical text, where the patterns are learnt automatically from examples. In particular we investigated how the performance of Ali Baba can be improved on four event extraction problems of the BioNLP task by a careful selection of the patterns of sentences. We described the effect of several pattern selection techniques for the gene expression event in detail and also provided result for three other event types. We showed that significant improvement can be achieved in terms of precision and F-score at a slight drop of the recall. Obviously, with a smaller pattern set, the runtime of the algorithms can be also largely decreased. The main advantages of our pattern filters compared to the similar methods presented in the literature are their simplicity and portability. The filters are very easy to implement and should be applicable at basically no cost to any pattern-based relation extraction method that works with automatically determined large pattern bases matched individually against new text, such as [10] or the method described in [33]. In addition, the proposed methodology offers a tool for tuning the precision-recall trade-off depending on the nature of the application task at hand.

## List of abbreviations

PPI: Protein-Protein Interaction; TM: Text Mining NLP: Natural Language Processing; NER: Named Entity Recognition; RE: Relation Extraction; POS: Parts of Speech; HR: Hit Rate; MDL: Minimum Description Length; FP: false positive; TP: true positive

## Competing interests

The authors declare that they have no competing interests.

## Authors' contributions

UL conceived of and supervised the project. QLN wrote the programs and performed all evaluations. DT participated in project's design and guided the evaluation. All authors contributed to writing and approved the manuscript.

## Authors' information

**Quang Long Nguyen **is a PhD student in the group of Ulf Leser. His work in text mining focuses on information extraction for life science applications, especially for Systems Biology.

**Domonkos Tikk **is an associate professor at Budapest University of Technology and Economics (on leave). Currently he is in Ulf Leser's group as an Alexander-von-Humboldt scholarship holder. His main research areas are text mining and recommendation systems.

**Ulf Leser **is a professor for Knowledge Management in Bioinformatics at the Humboldt-Universität in Berlin. His main topics of research are semantic data integration, text mining and biomedical data management.

## Supplementary Material

Additional file 1 **Effect of using different match thresholds evaluated on the development set**. Click here for file

Additional file 2 **Distribution of patterns according to length (number of tokens)**. Click here for file

Additional file 3 **F-score of subsets of patterns with identical length of *k *tokens on the development set**. Click here for file

Additional file 4 **Evaluation results of subsets with patterns of identical length on the development set**. Click here for file

## References

[B1] Cohen AM Hersh WR A survey of current work in biomedical text mining Brief Bioinform 2005 6 57 71 10.1093/bib/6.1.57 15826357

[B2] Zweigenbaum P Demner-Fushman D Yu H Cohen KB Frontiers of biomedical text mining: current progress Brief Bioinform 2007 8 358 375 10.1093/bib/bbm045 17977867 PMC2516302

[B3] Kao A Poteet S Natural Language Processing and Text Mining 2006 Springer Verlag

[B4] Chaussabel D Sher A Mining microarray expression data by literature profiling Genome Biol 2002 3 research0055 10.1186/gb-2002-3-10-research0055 12372143 PMC134484

[B5] Stapley BJ Benoit G Biobibliometrics: information retrieval and visualization from co-occurrences of gene names in Medline abstracts Pac Symp Biocomput 2000 529 540 10902200 10.1142/9789814447331_0050

[B6] Kuo C Chang Y Huang H Lin K Yang B Lin Y Hsu C Chung I Rich feature set, unification of bidirectional parsing and dictionary filtering for high F-score gene mention tagging Proceedings of the Second BioCreative Challenge Evaluation Workshop: Madrid, Spain 2007 Centro Nacional de Investigaciones Oncologicas (CNIO) 105 107

[B7] Kazama J Makino T Ohta Y Tsujii J Tuning support vector machines for biomedical named entity recognition Proceedings of Natural Language Processing in the Biomedical Domain: Philadelpia, PA, USA 2002 Association for Computational Linguistics 1 8 full_text

[B8] Tikk D Thomas P Palaga P Hakenberg J Leser U A comprehensive benchmark of kernel methods to extract protein-protein interaction from literature PLoS Compl Biology 2010 6 7 e1000837 10.1371/journal.pcbi.1000837 PMC2895635 20617200

[B9] Hakenberg J Plake C Royer L Strobelt H Leser U Schroeder M Gene mention normalization and interaction extraction with context models and sentence motifs Genome Biology 2008 Suppl 2 S14 10.1186/gb-2008-9-s2-s14 18834492 PMC2559985

[B10] Hao Y Zhu X Huang M Li M Discovering patterns to extract protein-protein interactions from the literature: Part II Bioinformatics 2005 21 3294 3300 10.1093/bioinformatics/bti493 15890744

[B11] Blaschke C Andrade MA Ouzounis C Valencia A Automatic extraction of biological information from scientific text: protein-protein interactions Proc Int Conf Intell Syst Mol Biol 1999 60 67 10786287

[B12] Saric J Jensen LJ Ouzounova R Rojas I Bork P Extraction of regulatory gene/protein networks from Medline Bioinformatics 2006 22 645 650 10.1093/bioinformatics/bti597 16046493

[B13] Fundel K Kuffner R Zimmer R RelEx - relation extraction using dependency parse trees Bioinformatics 2007 23 365 371 10.1093/bioinformatics/btl616 17142812

[B14] Ng SK Wong M Toward routine automatic pathway discovery from on-line scientific text abstracts Genome Inform Ser Workshop Genome Inform 1999 10 104 112 11072347

[B15] Blaschke C Hirschman L Valencia A Information extraction in molecular biology Brief Bioinform 2002 3 154 165 10.1093/bib/3.2.154 12139435

[B16] Cohen KB Verspoor K Johnson HL Roeder C Ogren PV Baumgartner WA White E Tipney H Hunter L High-precision biological event extraction with a concept recognizer Workshop on BioNLP: Shared Task at the Human Language Technology Conference (HLT); Boulder, CO, USA 2009 Association for Computational Linguistics 50 58

[B17] Rissanen J Modelling by shortest data description Automatica 1978 14 465 471 10.1016/0005-1098(78)90005-5

[B18] Kim J Ohta T Pyysalo S Kano Y Tsujii J Overview of BioNLP'09 shared task on event extraction Workshop on BioNLP: Shared Task at the Human Language Technology Conference (HLT); Boulder, CO, USA 2009 Association for Computational Linguistics 1 9

[B19] Plake C Schiemann T Pankalla M Hakenberg J Leser U Ali Baba: PubMed as a graph Bioinformatics 2006 22 2444 2445 10.1093/bioinformatics/btl408 16870931

[B20] Krallinger M Leitner F Rodriguez-Penagos C Valencia A Overview of the protein-protein interaction annotation extraction task of BioCreative II Genome Biol 2008 9 Suppl 2 S4 10.1186/gb-2008-9-s2-s4 18834495 PMC2559988

[B21] Björne J Heimonen J Ginter F Airola A Pahikkala T Salakoski T Extracting complex biological events with rich graph-based feature sets Workshop on BioNLP: Shared Task at the Human Language Technology Conference (HLT); Boulder, CO, USA 2009 Association for Computational Linguistics 10 18

[B22] Hakenberg J Mining Relations from the Biomedical Literature PhD thesis 2009 Humboldt-Universität zu Berlin

[B23] Kabiljo R Clegg A Sheperd A A realistic assessment of methods for extracting gene/protein interactions from free text BMC Bioinformatics 2009 10 233 10.1186/1471-2105-10-233 19635172 PMC2723093

[B24] Kim JD Ohta T Tsujii J Corpus annotation for mining biomedical events from literature BMC Bioinformatics 2008 9 10 10.1186/1471-2105-9-10 18182099 PMC2267702

[B25] Hastie T Tibshirani R Friedman J The elements of statistical learning 2001 Springer

[B26] Palaga P Nguyen L Leser U Hakenberg J High-performance information extraction with Ali Baba Proceedings of the 12th International Conference on Extending Database Technology (EDBT); St. Petersburg, Russia 2009 ACM 1140 1143 full_text

[B27] Buyko E Faessler E Wermter J Hahn U Syntactic simplification and semantic enrichment - trimming dependency graphs for event extraction Computational Intelligence in press

[B28] Xu F Uszkoreit H Li H A seed-driven bottom-up machine learning framework for extracting relations of various complexity Proceedings of the 45th Annual Meeting of the Associacion for Computational Linguistics (ACL); Prague, Czech Republic 2007 Association for Computational Linguistics 584 591

[B29] Hunter L Lu Z Firby J AB W Jr Johnson HL OpenDMAP: An open source, ontology-driven concept analysis engine, with applications to capturing knowledge regarding protein transport, protein interactions and cell-type-specific gene expression BMC Bioinformatics 2008 9 78 10.1186/1471-2105-9-78 18237434 PMC2275248

[B30] Björne J Ginter F Pyysalo S Tsujii J Salakoski T Complex event extraction at PubMed scale Bioinformatics 2010 26 12 i382 390 10.1093/bioinformatics/btq180 20529932 PMC2881365

[B31] Lease M Charniak E Dale R, Wong KF, Su J, Kwong OY Parsing Biomedical Literature Proceedings of the Second International Joint Conference on Natural Language Processing (IJCNLP'05); Jeju Island, Korea 2005 Berlin-Heidelberg; Springer 58 69

[B32] Giuliano C Lavelli A Romano L Exploiting shallow linguistic information for relation extraction from biomedical literature Proceedings of the 11th Conference of the European Chapter of the Association for Computational Linguistics (EACL): Trento, Italy 2006 Association for Computational Linguistics 401 408

[B33] Schneider G Kaljurand K Rinaldi F Detecting protein-protein interactions in biomedical texts using a parser and linguistic resources Proceedings of the International Conference on Computational Linguistics and Intelligent Text Processing 2009 Mexico City, Mexico 406 417 full_text

