# Supplementary figures and images for: Simple tricks for improving pattern-based information extraction from the biomedical literature
Source: J Biomed Semantics. 2010 Sep 24;1:9. doi: 10.1186/2041-1480-1-9 (PMC2955645; doi:10.1186/2041-1480-1-9)

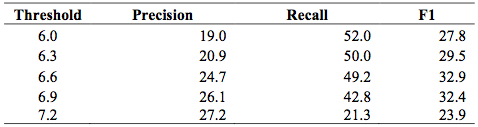

Supplement: Additional file 1 — Effect of using different match thresholds evaluated on the development set. [file 2041-1480-1-9-S1.PNG]

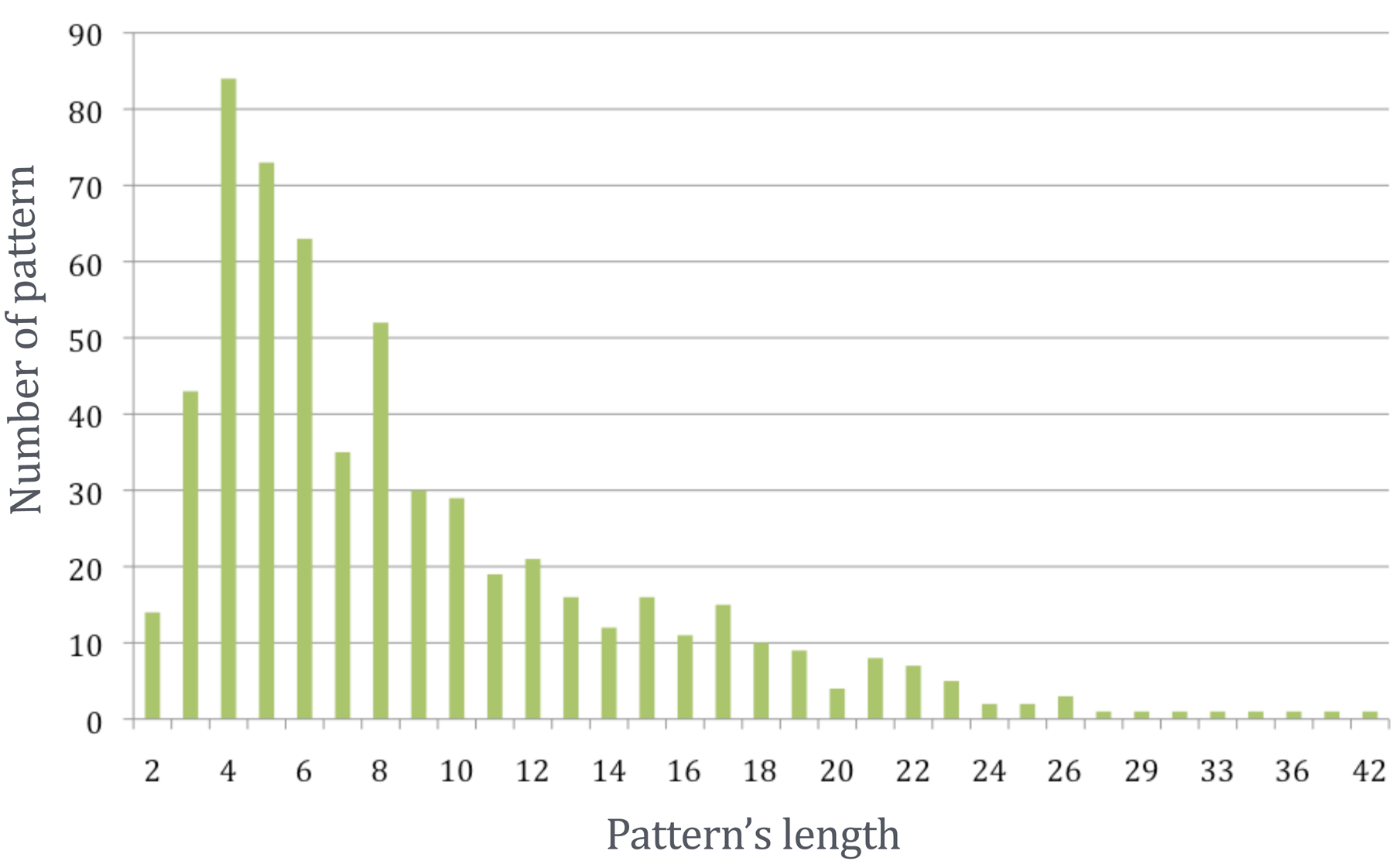

Supplement: Additional file 2 — Distribution of patterns according to length (number of tokens). [file 2041-1480-1-9-S2.PNG]

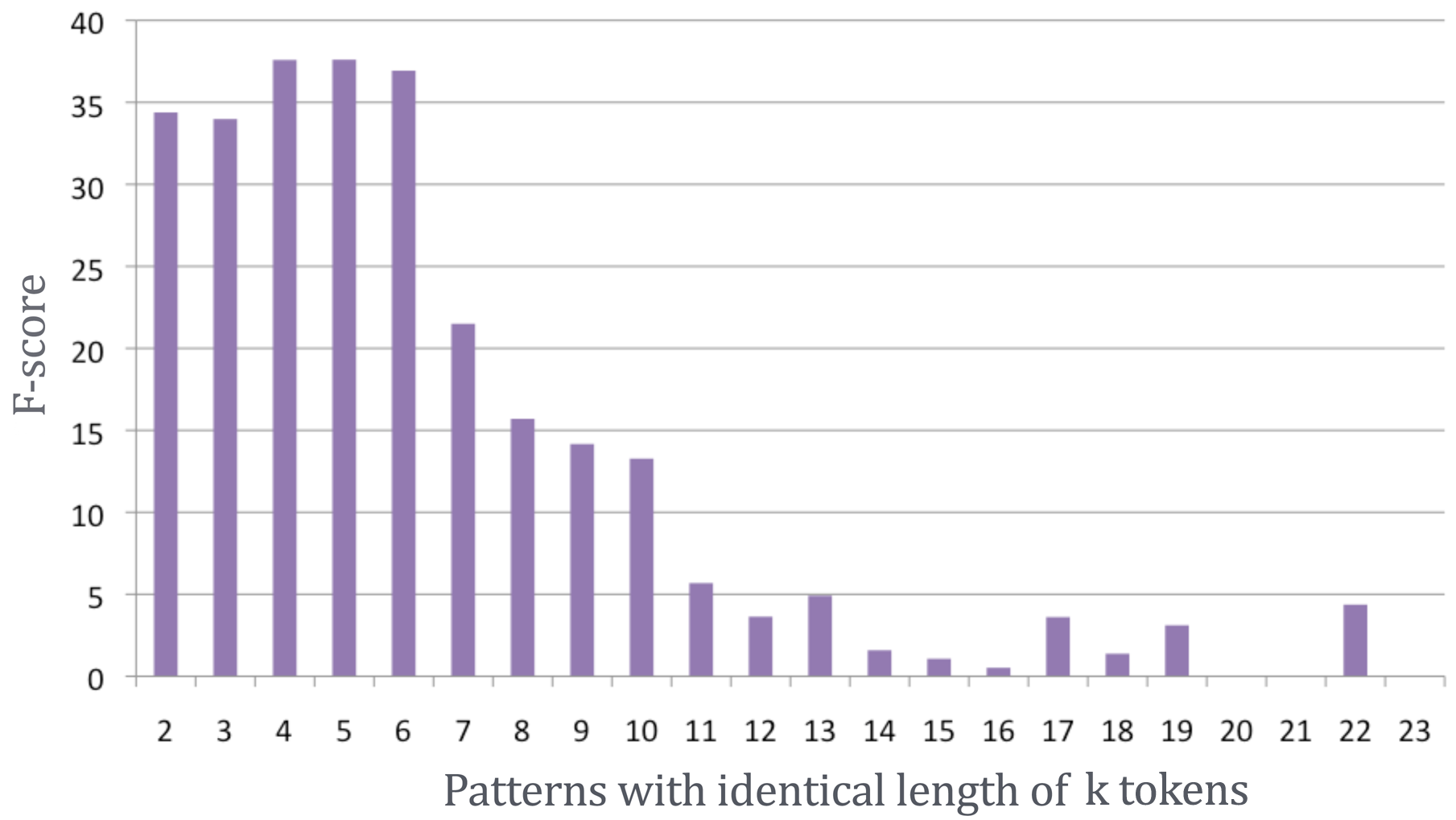

Supplement: Additional file 3 — F-score of subsets of patterns with identical length of k tokens on the development set. [file 2041-1480-1-9-S3.PNG]

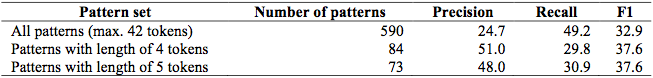

Supplement: Additional file 4 — Evaluation results of subsets with patterns of identical length on the development set. [file 2041-1480-1-9-S4.PNG]
